# Development of a High-Throughput Urosepsis Mouse Model

**DOI:** 10.3390/pathogens12040604

**Published:** 2023-04-15

**Authors:** Roman Herout, Sreeparna Vappala, Sarah Hanstock, Igor Moskalev, Ben H. Chew, Jayachandran N. Kizhakkedathu, Dirk Lange

**Affiliations:** 1 The Stone Centre at Vancouver General Hospital, Department of Urologic Sciences, University of British Columbia, Vancouver, BC V6H 3Z6, Canada; roman.herout@ukdd.de (R.H.); shanstock@prostatecentre.com (S.H.); ben.chew@ubc.ca (B.H.C.); 2 Department of Urology, University Hospital Carl Gustav Carus, Technische Universität Dresden, 01307 Dresden, Germany; 3 Centre for Blood Research, Life Science Institute, The University of British Columbia, Vancouver, BC V6T 1Z3, Canada; sreeparnav@hotmail.com (S.V.); jay@pathology.ubc.ca (J.N.K.); 4 Department of Pathology and Laboratory Medicine, The University of British Columbia, Vancouver, BC V6T 1Z3, Canada; 5 Vancouver Prostate Centre, The University of British Columbia, Vancouver, BC V6H 3Z6, Canada; imoskalev@prostatecentre.com; 6 School of Biomedical Engineering, The University of British Columbia, Vancouver, BC V6T 1Z3, Canada

**Keywords:** urosepsis, mouse model, catheter-associated urinary tract infection

## Abstract

Murine sepsis models are typically polymicrobial, and are associated with high mortality. We aimed to develop a high-throughput murine model that mimics a slow-paced, monomicrobial sepsis originating from the urinary tract. A total of 23 male C57Bl/6 mice underwent percutaneous insertion of a 4 mm catheter into the bladder using an ultrasound-guided method, previously developed by our group. The following day, *Proteus mirabilis (PM)* was introduced percutaneously in the bladder in three groups: g1—50 µL 1 × 10^8^ CFU/mL solution (*n* = 10); g2—50 µL 1 × 10^7^ CFU/mL solution (*n* = 10); and g3 (sham mice)—50 µL sterile saline (*n* = 3). On day 4, mice were sacrificed. The number of planktonic bacteria in urine, adherent to catheters, and adherent to/invaded into the bladder and spleen was assessed. Cell-free DNA, D-dimer, thrombin–antithrombin complex (TAT), and 32 pro-/anti-inflammatory cytokines/chemokines were quantified in the blood. All mice survived the 4 day postinterventional period. Mean weight loss was 11% in g1, 9% in g2, and 3% in the control mice. Mean urine CFU counts were highest in group 1. All catheters showed high catheter-adhered bacterial counts. Of the infected mice, 17/20 had CFU counts in the splenic tissue, indicating septicemia. Plasma levels of cell-free DNA, D-dimer, and the proinflammatory cytokines IFN-γ, IL-6, IP-10, MIG, and G-CSF were significantly elevated in infected mice versus controls. We present a reproducible, monomicrobial murine model of urosepsis that does not lead to rapid deterioration and death, and is useful for studying prolonged urosepsis.

## 1. Introduction

Sepsis is defined as a life-threatening organ dysfunction caused by a dysregulated host response to infection (Sepsis-3 Consensus Definition) [1]. When the infection leading to sepsis originates in the urinary tract, the term urosepsis is used. Urosepsis accounts for approximately 25% of all adult sepsis cases [2]. It is predominantly monomicrobial, with studies showing rates of 81–94% of cases where only a single pathogenic bacterial species could be detected [3,4].

The underlying pathophysiological processes in sepsis are complex and can involve two specific phases: the systemic inflammatory response (SIRS) and the compensatory anti-inflammatory response (CARS) [5,6]. In SIRS, a proinflammatory cascade is activated as a manifestation of an excessive reaction of the innate immune system to an initial stimulus. This leads to the production of chemokines, cytokines, and other mediators, resulting in an even more exacerbated reaction (“cytokine storm”). This proinflammatory response is countered by an anti-inflammatory reaction (CARS), and the balance between these two opposing processes is crucial for survival. Recent evidence suggests that sepsis should therefore be seen as a dynamic and heterogeneous disease due to imbalances in the “inflammatory network” [7]. In addition to the inflammatory shock, abnormal activation of coagulation, thrombin generation, and disseminated intravascular coagulation (DIC) are also characteristics of sepsis, all contributing to organ damage and failure [8,9].

Over the last three decades, murine models of sepsis and their relation to the highly complex human pathophysiology have been the subject of debate. Several mouse models of sepsis and septic shock have been developed, including: 1) the endotoxemia model, where a toxic agent, lipopolysaccharide (LPS), or another pathogen recognition receptor (PRR) ligand is injected either intravenously or intraperitoneally; 2) the bacterial injection model, where live pathogens (bacteria or intestinal content) are injected intravenously, intraperitoneally, or in another compartment; and 3) the impairment of barrier tissue integrity model (the cecal ligation and puncture model and the colon ascendens stent peritonitis model) [6]. While all of these models are acceptable in the study of sepsis, they do not represent the natural development of sepsis from an initial infection, as they rely on the intravenous or intraperitoneal administration of bacteria or their products, often leading to the rapid deterioration of the animals. Furthermore, they do not mimic the sequence of events leading to urosepsis, which is another common form of sepsis.

Urosepsis accounts for almost a third of all sepsis cases, with mortality rates as high as 25–60% in special patient groups [2,10]. To date, no specific murine urosepsis model exists. As a result, there is a significant need for the development of a model to better be able to study the sequence of events leading to urosepsis, as well as novel interventions to prevent them. To this end, we developed an animal model that aims at mimicking sepsis originating from the urinary tract by modifying our established catheter-associated urinary tract infection (CAUTI) model, and provide a useful tool for further studies pertaining to urosepsis and therapeutic options.

## 2. Materials and Methods

### 2.1. Mice

All animal studies were approved by The University of British Columbia Animal Care Committee (Protocol A17-0297). A total of 23 male C57Bl/6 mice (Harlan^®^, Indianapolis, IN, USA) at 12 weeks of age, weighing between 20 and 25 g, were used. The mice were housed at our facility at a temperature of 23 ± 1 °C (relative humidity of 50 ± 10%) with 12 h of alternating lighting. Mice were housed in cages in groups of 2–4 animals per cage, and study animals of different study groups could not be housed together to avoid cross-contamination. During the study period, food and drink were provided ad libitum.

### 2.2. Bacteria

To induce sepsis, *Proteus mirabilis (PM)* strain HI4320, which was isolated from the urine of a female nursing home patient with an indwelling urinary catheter, was used [11,12]. We used *Proteus mirabilis*, as it frequently causes catheter-associated urinary tract infections, and is the major culprit for infection stone formation and catheter blockage and failure. Elderly, immobile patients with indwelling catheters are at high risk of CAUTI, with subsequent struvite stone formation that can cause sepsis in this patient group [13].

*PM* was cultured from freezer stocks (−80 °C) in fresh LB media (10 g/liter tryptone, 5 g/liter yeast extract, 0.5 g/liter NaCl) and incubated at 37 °C overnight. The following day, cultures were subcultured in fresh LB media and left overnight in the same conditions. The overnight culture was plated on LB agar plates, as well as resuspended to an optical density of 0.5 at 600 nm. The subculture and the final solution of PBS with bacteria were plated as well, to confirm the exact number of bacteria instilled into the animals.

### 2.3. Animal Surgery

#### 2.3.1. Catheter Modification

To create a catheter piece that could be introduced percutaneously into the urinary bladder, we used commercially available 24G angiocatheters (Terumo Surflash^®^, Tokio, Japan; Polyurethane I.V. Catheter 24 Gauge × 3/4”, Cat. No. SRFF2419). Under aseptic conditions, we temporarily removed the polyurethane plastic tip from the needle and a 4 mm piece was cut off using a sterile blade. The cut off tip was then reassembled back onto the needle tip. Prior to the experiment, all catheters were gas sterilized with ethylene oxide to further ensure sterility and to avoid contamination.

#### 2.3.2. Mouse Experiments

After induction of anesthesia with 3% isoflurane, a 4 mm piece of a modified 24G catheter (as described above) was implanted into the mouse bladder under ultrasound guidance (Vevo 770^®^ High-Resolution Imaging System), as previously described [14]. The day following catheter implantation, all mice underwent anesthesia under the same conditions as above. *Proteus mirabilis* was introduced percutaneously in the bladder in 3 groups: group 1–50 µL of a 1 × 10^8^ CFU/mL solution (*n* = 10); group 2–50 µL of a 1 × 10^7^ CFU/mL solution (*n* = 10); and group 3 (sham mice) –50 µL of sterile saline (*n* = 3). To prevent premature micturition, and to allow for the bacteria to attach to the catheter, mice were kept under anesthesia at 1% isoflurane for an additional hour after instillation of the bacterial solution. After the intervention, mice were monitored closely, and on day 4 all mice were sacrificed: anesthesia was induced with 3% isoflurane and urine was extracted percutaneously under aseptic conditions and ultrasound guidance. Urine samples were then serially diluted and plated on LB agar plates for colony forming unit (CFU) counts. Mouse bladders were halved under aseptic conditions, the catheter extracted, washed thrice with sterile PBS, and transferred to a tube with sterile PBS. Catheter samples were then sonicated at 50/60 Hz for 10 min in an ultrasonic water bath (No. 21811-820, VWR^®^) to obtain detachment of the adherent bacteria from the catheter into the PBS. The sample tubes were then vortexed at high speed for 30 s to enable uniform distribution throughout the medium. PBS–bacterial solutions were then serially diluted and plated on LB agar plates. After incubation at 37 °C, the CFUs on each plate were counted.

To quantify the bacteria that had adhered and invaded the mouse bladder, adhesion and an antibiotic protection assay were performed [15]. After the bladders were harvested, they were aseptically bisected and washed in sterile PBS. Half of the bladder was placed in sterile DMEM + 10% FBS for the adhesion CFU counts. The other bladder half underwent an antibiotic protection assay and was placed in sterile DMEM + 10% FBS + 100 μg/mL penicillin–streptomycin. Both bladder halves were then incubated at 37 °C for 1 h on a gentle shaker. Each sample was washed with sterile PBS three times and placed inside a tissue homogenizer tube containing fresh sterile PBS. All bladder samples were tissue homogenized using the Precellys 24 (Bertin technologies, Montigny-le-Bretonneux, France), serially diluted, and plated on LB agar plates. Likewise, the spleen was harvested upon necropsy, washed in sterile PBS, and homogenized using the Precellys 24. The homogenate was serially diluted and plated on LB agar plates. All agar plates were incubated overnight at 37 °C until bacterial colonies were visible, and CFU counts between 2 and 50 colonies were documented.

Blood was collected via cardiac puncture. Analysis was available for 22 mice in total, as one sample could not be analyzed. Citrated blood was immediately spun down at 1200 g for 10 min to remove red blood cells, followed by centrifugation at 10,000× *g* for 10 min to remove any residual cells. DNA concentration was measured using a Quant-iT™ PicoGreen™ dsDNA Assay Kit according to the manufacturer’s instructions. TAT complex was measured using the Mouse Thrombin–Antithrombin (TAT) Complexes ELISA Kit according to the manufacturer’s instructions. The cytokine profile in the plasma was analyzed using the Mouse Cytokine/Chemokine 31-Plex Discovery Assay^®^ Array (MD31) service from Eve technologies. Levels of the following cytokines were measured: Eotaxin, G-CSF, GM-CSF, IFN-γ, IL-1α, IL-1β, IL-2, IL-3, IL-4, IL-5, IL-6, IL-7, IL-9, IL-10, IL-12p40, IL-12p70, IL-13, IL-15, IL-17, IP-10, KC, LIF, LIX, MCP-1, M-CSF, MIG, MIP-1α, MIP-1β, MIP-2, RANTES, TNF-α, and VEGF.

### 2.4. Statistical Analysis

The statistical analysis war performed with GraphPad Prism (version 8) software (Dotmatics, Boston, MA, USA). The Welch analysis of variance tests and the Dunnett’s T3 multiple comparison tests were used to compare CFU counts, immunoassay concentrations, as well as plasma concentrations of cell-free DNA, D-dimer, and TAT complex. All tests were two tailed, and a P value of less than 0.05 was considered significant.

## 3. Results

### 3.1. Clinical Assessment of Animals

After percutaneous injection of the bacterial solution into the urinary bladder, mice were closely monitored for clinical symptoms of sepsis. All mice lost weight during the study period. Mean weight loss was 11% in group 1 (50 µL of 1 × 10^8^ solution), 9% in group 2 (50 µL of 1 × 10^7^ solution), and 3% in the control mice (sterile saline injection).

### 3.2. CFU Counts

**Figure 1:**
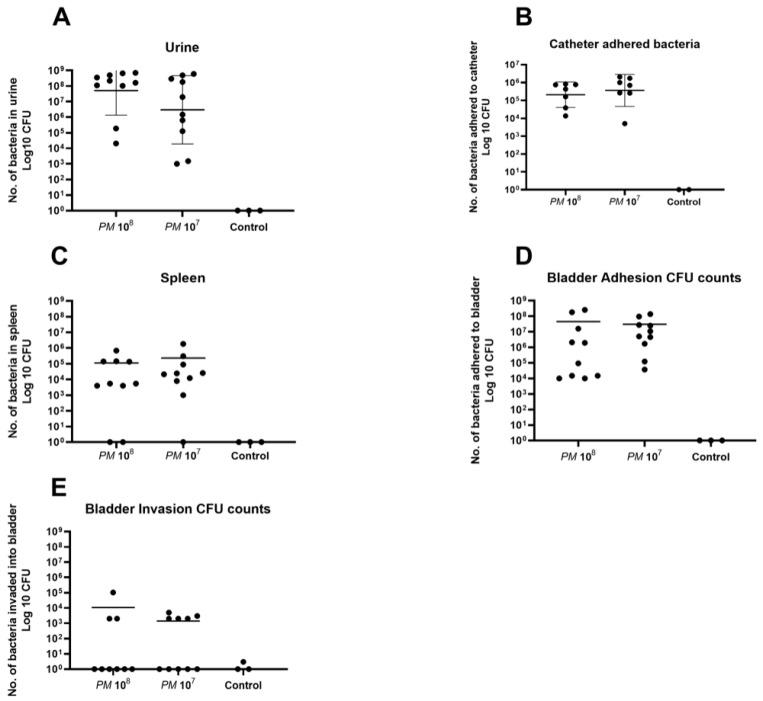
Scattered dot plot with the geometric mean and geometric SD showing CFU counts of *Proteus mirabilis*. Each dot represents the total CFU recovered from a single animal. (**A**) Elevated CFU counts of *PM* in urine of both groups with no statistical difference between the two infected groups. All urine samples of control mice were negative for *PM*. (**B**) High CFU counts of *PM* for both infected groups with no statistical difference. All catheters of control mice showed no bacterial growth. (**C**) High CFU counts in splenic tissue in eight mice of group 1 (two negative) and nine mice in group 2 (one negative). No bacterial growth was detected in splenic tissue of control mice. (**D**) High CFU counts of bacteria that adhered to bladder urothelium for both infected groups with no statistical difference between them. All controls were negative for *PM*. (**E**) Invasion of *PM* into bladder tissue in three mice of group 1, and in five mice of group 2 on day 4 of the experiment. No bacterial growth was seen in control mice.

A total of 23 urine samples were obtained under anesthesia via sterile puncture and aspiration under ultrasound guidance. Mean CFU counts in urine of mice infected with *PM* wild-type were 2.8 × 10^8^ in group 1 (*n* = 10), 1.6 × 10^8^ in group 2 (*n* = 10), and all control urine samples were negative for bacterial growth (Figure 1A).

In total, 16 catheters could be retrieved in the urinary bladder, as 6 catheters were lost upon necropsy. Mean catheter adhered bacterial CFU counts for group 1 (*n* = 7) were 4.2 × 10^5^, 8.6 × 10^5^ for group 2 (*n* = 7), and all controls (*n* = 2) were negative for bacterial growth (Figure 1B).

*PM* could be detected in the spleen of 17 of the 20 infected mice (85%). Mean CFU counts in splenic tissue were 1.1 × 10^5^ for group 1 (*n* = 8), 2.3 × 10^5^ for group 2 (*n* = 9), and all controls were negative for *Proteus mirabilis* (Figure 1C). CFU counts for *Proteus mirabilis* adhered to bladder tissue were, on average, 4.5 × 10^7^ for group 1 (*n* = 10), 3.0 × 10^7^ for group 2 (*n* = 10), and controls (*n* = 3) did not show any growth on agar plates after overnight incubation (Figure 1D). Invasion of bacteria into bladder cells was seen in three animals of group 1 (30% of *n* = 10) with a mean CFU count of 3.6 × 10^4^, in five animals of group 2 (50% of *n* = 10) with a mean of 2.8 × 10^3^, and *Proteus mirabilis* could not be detected in the homogenized bladder tissue of the three control mice (Figure 1E).

### 3.3. Plasma Levels of Cell-Free DNA, D-Dimer, and TAT Complex

**Figure 2:**
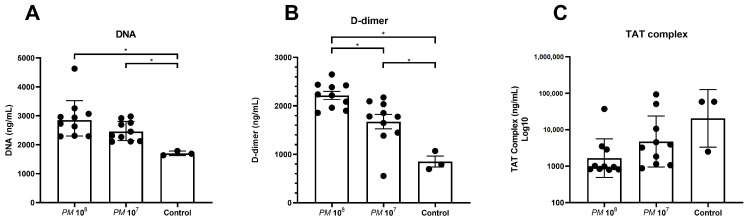
Scattered dot plot with the geometric mean and geometric SD showing plasma levels of cell-free DNA, D-dimer, and TAT complex in infected and control mice. Each dot represents the plasma level of the investigated substance from a single animal. Statistically significant differences are indicated by an asterisk (* *p* < 0.05). (**A**) Cell-free DNA in plasma with statistically significant higher levels in infected mice when compared to controls. (**B**) Statistically higher levels of D-dimer in plasma of infected mice when compared to controls, but also significantly higher levels in group 1 when compared to group 2. (**C**) Highest levels of TAT complex in controls when compared to infected mice, but no statistically significant difference was seen between the groups.

Levels of cell-free DNA were significantly elevated in plasma of infected mice versus controls, as shown in Figure 2A. Levels of D-dimer were significantly higher in infected mice when compared to control mice, but mice in group 1 (10^8^) also showed significantly higher levels when compared to group 2 (10^7^).

### 3.4. Plasma Levels of Chemokines and Cytokines

**Figure 3:**
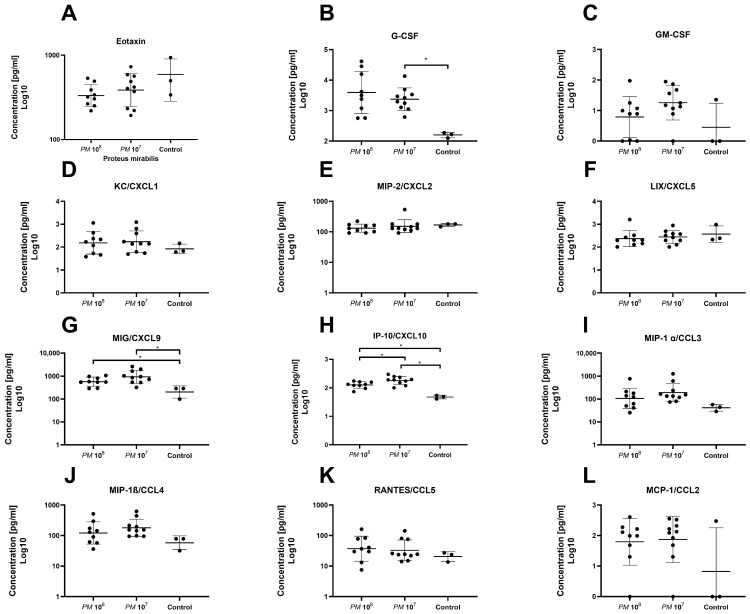
Plasma levels of chemokines of infected and control mice. Each dot represents the plasma level of the investigated substance from a single animal. Statistically significant differences are indicated by an asterisk (* *p* < 0.05). (**A**) Highest levels of Eotaxin were seen in controls; group 2 showed slightly higher levels than group 1, but no statistical significance was seen. (**B**) Higher levels of G-CSF were observed in infected mice when compared to controls, with a significant difference between group 2 and controls. (**C**) Higher levels of GM-CSF were observed in infected animals with no significant difference. Levels of KC/CXCL1 (**D**), MIP-2/CXCL2 (**E**), and LIX/CXCL5 (**F**) were similar between groups. (**G**) Levels of MIG/CXCL9 were significantly elevated in infected animals vs. controls. (**H**) Levels of IP-10/CXCL10 were significantly elevated in infected animals when compared to control, but also significantly higher in group 2 when compared to group 1. Plasma levels of MIP-1 α/CCL3 (**I**), MIP-1β/CCL4 (**J**), RANTES/CCL5 (**K**), and MCP-1/CCL2 (**L**) were similar among groups.

**Figure 4:**
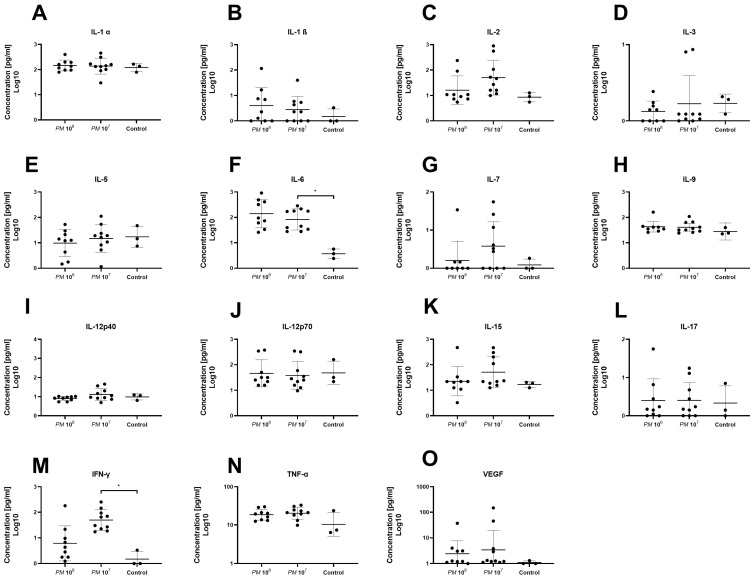
Plasma levels of proinflammatory cyto- and chemokines of infected and control mice. Each dot represents the plasma level of the investigated substance from a single animal. Statistically significant differences are indicated by an asterisk (* *p* < 0.05). Levels of IL-1α (**A**) and IL-1β (**B**) were similar among groups. (**C**) Levels of IL-2 were higher in group 2 vs. group 1 and controls, but not statistically significant. Levels of IL-3 (**D**) and IL-5 (**E**) were similar among groups. (**F**) Levels of IL-6 were significantly higher in group 2 when compared to controls. (**G**) Highest levels of IL-7 were seen in group 2 without significance. Levels of IL-9 (**H**), IL-12p40 (**I**), IL-12p70 (**J**), IL-15 (**K**), and IL-17 (**L**) were similar among groups. (**M**) Plasma levels of IFN-γ were significantly elevated in group 2 when compared to group 1 and controls. Levels of TNF-α (**N**) and VEGF (**O**) were similar among groups.

**Figure 5:**
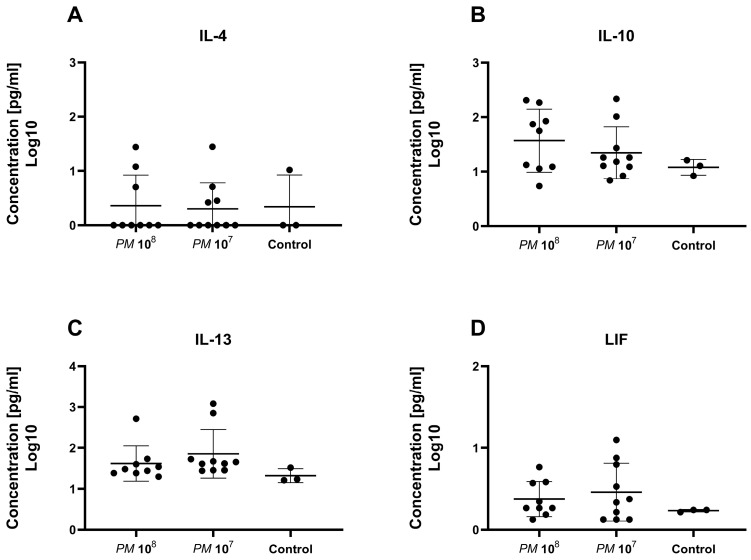
Scattered dot plot with the geometric mean and geometric SD showing plasma levels of anti-inflammatory cytokines. Each dot represents the plasma level of the investigated substance from a single animal. We did not observe a statistically significant difference in plasma levels of IL-4 (**A**), IL-10 (**B**), IL-13 (**C**), and LIF (**D**) between the groups.

A total of 32 chemo- and cytokines were measured in plasma to assess whether sepsis was induced in the mice intravesically infected with high doses of *Proteus mirabilis*. Proinflammatory cytokines TNF-α, IL-1β, IL-6, and MCP-1 were all elevated in infected mice when compared to controls. In total, levels of 26 of the 32 chemo- and cytokines were higher in both infected mice groups when compared to healthy control animals. However, statistically significant differences were only seen for G-CSF, MIG, IP-10, IL-6, and IFN-γ. Proinflammatory chemo- and cytokines are depicted in Figure 3 and Figure 4. Anti-inflammatory cytokine levels are shown in Figure 5.

**Figure 6:**
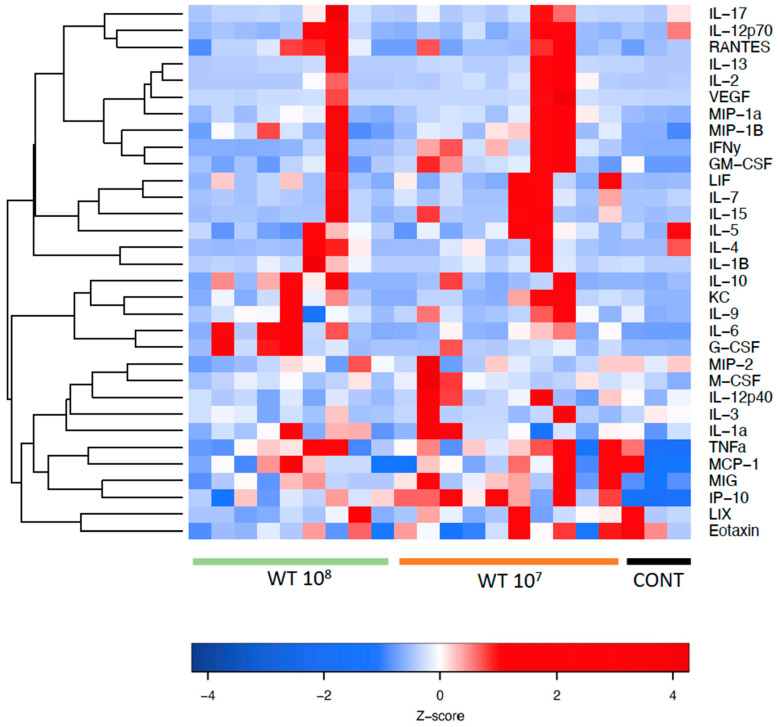
Heatmap showing the panel of cytokines and chemokines in mouse plasma. The color scheme is red and blue, where red indicates high relative levels and blue indicates low relative levels.

Figure 6 shows a heatmap with the panel of cyto- and chemokines that were investigated in mouse plasma. The red color indicates high relative levels and the blue color indicates low relative levels of the investigated cyto- and chemokines.

## 4. Discussion

We present data of our exploratory study to establish a urosepsis mouse model. To date, no mouse sepsis model is available where the infection originates in the urinary tract, hence it was our effort to create a monomicrobial murine model of urosepsis that mimics a slower-paced sepsis, as compared to established models, such as the CLP method.

We demonstrated a dissemination of bacteria, initially injected percutaneously into the urinary bladder of mice, into the bloodstream via detection of bacteria in the spleen, indicating septicemia. By percutaneously inserting a 4 mm catheter piece under ultrasound guidance into the urinary bladder, the subsequently injected bacteria found a nidus on which to attach and grow a biofilm. Hence, clearance of bacteria is markedly impeded and a pronounced urinary tract infection is established. All infected mice had high CFU counts of *PM* in urine, which was collected under sterile conditions. All catheters that could be obtained from infected mouse bladders showed high CFU counts of attached bacteria, indicative of a mature and well-established biofilm acting as a nidus for the perpetual infection. Additionally, high numbers of bacteria attached to the bladder were found in the adhesion assay of homogenized bladder tissue and, as in previous experiments performed by our group, we found invasion into bladder tissue in some of the infected animals [16].

To further verify that infected mice were septic, and to prove the validity of the model, we measured a set of 32 cytokines in the plasma of the study animals. Levels of IFN-γ and IL-6 were significantly elevated in infected mice when compared to controls. In total, 26 of the 32 cytokines were nonsignificantly elevated in the mice with urosepsis when compared to sham mice. Levels of anti-inflammatory cytokines, IL-4, IL-10, IL-13, and leukemia inhibitory factor (LIF) were similar among all three groups.

As hypercoagulation and DIC are hallmarks of sepsis, we subsequently quantified cell-free DNA, D-dimer, and thrombin–antithrombin (TAT) complex in the plasma of the cohort. During sepsis, neutrophils, as the most abundant innate immune effector cells, are recruited to fight the pathogens causing the infection. Therefore, neutrophil extracellular traps (NETs), which are extracellular, web-like structures composed of DNA and proteins, are released [17]. To discriminate between septic mice and controls, we measured cell-free DNA in the plasma of the mice, and significantly higher levels of cell-free DNA were seen in the infected mice when compared to the control group. D-dimer is a degradation product of fibrin clot created during fibrinolysis, and is a well-established, very sensitive clinical marker of coagulation [18]. D-dimer was significantly elevated in septic mice when compared to control mice. The thrombin–antithrombin (TAT) complex is formed to limit coagulation, and represents a very sensitive serum marker [19]. For the TAT complex, levels were higher in the control group versus the infected mice, which is a finding that is hard to explain. However, it could be attributed to incorrect collection, as we punctured the heart with a needle through the skin, which may contain activators of clotting-like tissue factor that promote TAT complex formation and thrombin generation, as well as the short half-life of the molecule of only 15 min.

To date, several murine models of sepsis are well-established; however, all models have major drawbacks, and none is based on an infection of the urinary tract.

In the endotoxemia model, endotoxins such as LPS are used to induce a sepsis-like condition. The major concern with the endotoxemia model is the injection of a chemical mediator instead of a viable pathogen, which might not reproduce the complex pathophysiologic response to viable pathogens [6]. Studies have also shown that mice require higher doses of LPS to exhibit symptoms, and are generally less sensitive to LPS when compared to humans [20].

The cecal ligation and puncture (CLP) and the colon ascendens stent peritonitis (CASP) models are so called “Host-barrier disruption models”, as they are defined by a leakage through a barrier that, under physiologic conditions, protects sterile compartments from pathogens [6]. In the CLP model, the appendix is ligated after laparotomy and punctured with a needle to allow feces to enter the peritoneal cavity. In the CASP model, a stent is inserted in the ascending colon, which facilitates fecal matter to continuously enter the peritoneal cavity. Both models induce a very rapid host response, and ultimately a foudroyant sepsis. A dramatic increase in serum levels of proinflammatory cytokines, such as IL-6 and TNF-α, can be observed within hours [21,22]. As Kingsley and Bhat outlined in their review article, the two models complement each other in their responses; in the CLP model, generally only proinflammatory cytokines are elevated, whereas in the CASP model, a rapid elevation of both pro- and anti-inflammatory cytokines can be detected [23]. However, the rapid onset of sepsis with a high mortality, as well as the fact that the induced sepsis is polymicrobial, do not pose them as ideal models for urosepsis.

Lastly, the bacterial infection model represents a model that provides crucial insights into mechanism of the host response. However, is has been critiqued for its significant interlaboratory variability (growth and quantification of bacteria prior to experiment), dependance on specific pathogens as well as specific host responses, and the fact that a potentially life-saving medication used in humans is withheld in animals, and could distort the potential benefits of tested therapeutic agents. Typically, high numbers of bacteria are needed, and hosts are frequently able to clear pathogens of the infected compartments.

Our model can overcome some of the above-mentioned limitations of the bacterial infection models: First, by implanting a 4 mm catheter piece in the urinary bladder, we actively insert a nidus for bacteria to grow and replicate on, hence massively impeding natural clearance of the pathogen. Therefore, a constant source of pathogens is established that mimics infection in sepsis patients. Secondly, our model induces a slow-paced sepsis with gradual deterioration of the animal’s overall health over multiple days, instead of leading to death within hours when compared to the CLP model. This is especially useful when drugs are tested that need some time to reach a steady state in the plasma to exert therapeutic effects.

Li et al. showed that plasma levels of IL-6, TNF-α, MIP-1α, MIP-1β, and MIP-2 spiked after 8 h post-CLP surgery, and then decreased steadily over time. However, levels were still elevated after 48 h when compared to the baseline value [24]. This is in line with our findings, where levels of 26 out of 28 proinflammatory chemo- and cytokines were elevated in infected mice when compared to controls. However, statistical significance was only reached for G-CSF, IFN-γ, IL-6, IP-10, and MIG. All 23 animals survived the 48 h period postintervention, with a weight loss of 11% in group 1, 9% in group 2, and 3% in the sham group. The fact that mice in the control group lost weight after implantation of the catheter shows that the foreign body most likely induces an inflammation of the bladder, and leads to irritative voiding symptoms that subsequently impacts overall health and food consumption. On ultrasound, the bladder walls were thickened and appeared inflamed.

### 4.1. Strengths

We present a murine urosepsis model that is monomicrobial and mimics a slow-paced sepsis. The initiating pathogen is primarily located in the urinary tract and spreads in a hematogenous manner throughout the body. By inserting a 4 mm catheter piece we actively implant a nidus for bacterial attachment and, therefore, facilitate intracorporeal replication and subsequent dissemination of the pathogen. Due to the relatively slow onset of symptoms, the clinical sepsis has time to develop, and our model yields a high survival rate.

### 4.2. Limitations

As with all sepsis models, we must address some limitation: First, the absence of comorbidities during sepsis development is something that needs to be considered. However, this represents a major problem for all animal models of sepsis, and is not specific for the one presented in this work. Secondly, the lack of supportive care in a standardized fashion, as would be administered to humans (no vasopressors, ventilators, etc.), can never be exactly reproduced in an animal model. Additionally, pro- and anti-inflammatory cytokines were only measured once at the endpoint of the study, so we do not have levels over time, which could give us a better insight in the various phases of the sepsis. Another drawback of our method is the need for an ultrasound machine dedicated to animal work with a high frequency ultrasound probe. In addition, there is a learning curve for the percutaneous insertion of the catheter piece. However, with proper training, we consider this method to be easily reproducible and fast.

## 5. Conclusions

We present an easily reproducible murine model of urosepsis that mimics a slow-paced sepsis. The presented model can overcome some of the drawbacks experienced with established sepsis models, and can help in testing drugs that have to be administered over longer periods of time, as most animals will survive at least 48 h postinfection.

## Data Availability

The data presented in this study are available upon reasonable request from the corresponding author via dirk.lange@ubc.ca.
